# Physiologically-based pharmacokinetic modeling and simulation for initial dose optimization of levetiracetam in pediatrics

**DOI:** 10.3389/fphar.2025.1678960

**Published:** 2025-12-04

**Authors:** Julia Macente, Rodolfo Hernandes Bonan, Edilainy Rizzieri Caleffi-Marchesini, Leonardo Régis Leira Pereira, Priscila De Freitas Lima, Pieter Annaert, Karel Allegaert, Andrea Diniz

**Affiliations:** 1 Drug Delivery and Disposition, Department of Pharmaceutical and Pharmacological Sciences, KU Leuven, Leuven, Belgium; 2 BioNotus CommV, Niel, Belgium; 3 Pharmacokinetics and Biopharmaceutical Laboratory (PKBio), Department of Pharmacy, State University of Maringa, Maringa, Brazil, Department of Pharmacy, State University of Maringá, Maringá, Paraná, Brazil; 4 Faculty of Pharmaceutical Sciences of Ribeirão Preto, University of São Paulo, Ribeirão Preto, São Paulo, Brazil; 5 Barão de Mauá University Center, Ribeirão Preto, São Paulo, Brazil; 6 Clinical Pharmacology and Pharmacotherapy, Department of Pharmaceutical and Pharmacological Sciences, KU Leuven, Leuven, Belgium; 7 Department of Development and Regeneration, KU Leuven, Leuven, Belgium; 8 Department of Hospital Pharmacy, Erasmus University Medical Center, Rotterdam, Netherlands

**Keywords:** levetiracetam, PBPK modeling, pediatric population, dose optimization, epilepsy

## Abstract

**Introduction:**

Optimizing levetiracetam (LEV) dosing in children is challenging due to high pharmacokinetic variability, which often necessitates empirical dose titration. This study aimed to develop and verify a physiologically-based pharmacokinetic (PBPK) modeling and simulation to guide and optimize initial LEV dose selection in pediatric patients.

**Methods:**

A whole-body PBPK model for LEV was developed and verified in adults, then scaled and verified in a pediatric population (0.5–12 years). This model was used to simulate various dosing regimens. Subsequently, a multivariate linear regression (MLR) analysis correlated key covariates (dose, regimen, body weight, and glomerular filtration rate) with simulated steady-state peak (C_max_) and trough (C_tr_) concentrations to create a practical dosing tool.

**Results:**

The MLR model successfully explained over 90% of the variance (R^2^ > 0.9) between covariates and simulated plasma concentrations. For a twice-daily regimen, daily doses of 40–60 mg/kg were required to achieve concentrations within a target therapeutic window (*e.g.,* C_max_ of 20–46 mg/L). A three-times-daily regimen allowed for a broader effective dose range of 50–80 mg/kg/day, enabling higher total daily doses while maintaining C_max_ within a safe range.

**Conclusion:**

The combined PBPK-MLR approach provides a robust, data-driven framework to support rational first-dose prescriptions of LEV in children. This tool has the potential to accelerate therapeutic effects while enhancing treatment individualization. Prospective clinical validation is required to confirm the model predictive performance for drug exposure and, consequently, its impact on therapeutic efficacy.

## 1 Introduction

Levetiracetam is a widely used second-generation anticonvulsant approved for both adult and pediatric populations as a first-line or adjunctive treatment for various seizure types (Celdran de Castro et al., 2023, UCB Biosciences, 1999). It has a favorable pharmacokinetic profile, including near-complete oral absorption, low plasma protein binding (<10%), and linear kinetics, which generally make its pharmacologic response predictable (Patsalos, 2000). LEV is primarily eliminated renally, with approximately 66% of the dose excreted unchanged in the urine. Its minimal reliance on hepatic metabolism results in a low potential for drug-drug interactions, an advantage in polytherapy regimens commonly used in epilepsy management.

Despite these favorable properties, optimizing LEV dosing in the pediatric population remains challenging. Pediatric patients exhibit higher renal clearance per kilogram compared to adults, necessitating weight-based dosing strategies to achieve similar exposures (Glauser et al., 2002; Pellock JM et al., 2001). This, combined with the dynamic physiological changes occurring from infancy through adolescence, complicates dose selection and has led to widespread *off-label* use and dosing variability in clinical practice (Franco et al., 2016).

A further complication is the lack of a universally accepted therapeutic window for LEV. Although suggested therapeutic ranges for plasma concentrations have been proposed, the relationship between plasma levels and therapeutic response varies widely between individuals, making routine therapeutic drug monitoring (TDM) uncommon (Sinha et al., 2022). As a result, clinicians often rely on empirical dose titration, a time-consuming approach that may delay effective seizure control. Additionally, LEV has been associated with behavioral adverse events in children, which appear to be more related to the rate of dose escalation than the final maintenance dose. This suggests that reaching therapeutic levels quickly, without aggressive titration, could improve both efficacy and tolerability.

The application of physiologically-based pharmacokinetic (PBPK) modeling to levetiracetam (LEV) has been instrumental in addressing dosing challenges in pediatric populations. For instance, Shao et al. (2023) developed a PBPK model to optimize LEV dosing for Chinese children, finding that standard recommendations may lead to underexposure in this group. Similarly, a model by Maglalang et al. (2024) was extended to account for LEV disposition in infants and children with obesity, offering critical insights for these understudied populations.

While these models have advanced our understanding, there remains a gap in providing clinicians with a practical, quantitative framework to guide initial LEV across a broad pediatric age range. There is a clear need to move beyond empirical titration toward a more rational strategy that safely accelerates the time to therapeutic drug concentrations. Key unanswered questions, such as the optimal frequency of administration (*e.g.,* twice-daily vs. three times a day) and the justification for more assertive starting doses are critical to improve clinical outcomes.

All therapeutic window recommendations are related to the minimal concentration in the body (C_tr_), mainly because to get blood samples to quantify, experimentally the maximum concentration (C_max_) is an improbable success. However, for many drugs, C_max_ is directly linked to concentration-dependent side effects and toxicity. Relying solely on trough levels could therefore mask potential harm to the patient, making the estimation or avoidance of high peak concentrations a critical aspect of safe and effective therapy, especially for drugs with a narrow therapeutic index.

Therefore, the aim of this study was to develop and verify a whole-body PBPK model for LEV in a pediatric population aged 6 months to 12 years. The model was designed to evaluate different dosing strategies and provide a scientific rationale to support initial dose selection, with the goal of safely and rapidly achieving therapeutic concentrations.

## 2 Methodology

### 2.1 Adult PBPK model development

A whole-body PBPK model for LEV was first developed in healthy adult volunteers using GastroPlus software version 9.8 (Simulations Plus, Lancaster, CA). Drug-specific physicochemical and pharmacokinetic parameters (*e.g.,* logP, pKa, molecular weight, solubility, fraction unbound in plasma, intestinal permeability, and blood-to-plasma ratio) were obtained from the literature and used as model inputs (Table 1). Intestinal permeability (25.5 × 10^−6^ cm/s) was used to define the absorption process. The volume of distribution was predicted using the Poulin and Theil model (Poulin & Theil, 2009). The total clearance (CL_total_) is known to occur via two main routes: approximately 66% through glomerular filtration and 34% through esterase-mediated metabolism (Patsalos, 2000). However, the specific metabolic enzymes responsible for this non-renal clearance remain unidentified. Some studies suggest the involvement of serum esterase (Di, 2019) or hepatic esterase activity (Boberg et al., 2017; Wang et al., 2016), but no consensus has been reached regarding the exact enzymatic pathway. Given this uncertainty, it was assumed in this study that 34% of total clearance is attributed to non-renal pathways (CL_NR_), without specifying the enzyme system, while the remaining 66% was attributed to net renal clearance, accounting for both glomerular filtration and tubular reabsorption.

**TABLE 1:** Drug-dependent parameters for Levetiracetam PBPK model development.

Parameter (Units)	Value	References
MW (g/mol)	170.21	Drugbank. Levetiracetam (2021) ^a^
logP	−0.67	Mangelings et al. (2006)
pKa	15.74	Mangelings et al. (2006)
Solubility (mg/mL)	104.0	Petruševska et al. (2015)
Specific intestinal permeability (10^-6^ cm/s)	25.5	Masungi et al. (2008), Petruševska et al. (2015)
Fup (%)	90	Patsalos (2000), Strolin Benedetti et al. (2003), Drugbank. Levetiracetam (2021) ^a^
B/P	1.3	Strolin Benedetti et al. (2003)
Kp	0.73	Calculated, Poulin and Theil (2002)
Adult		
*V*_ss_ (L)	37.02	Calculated, Poulin and Theil (2002)
Cl_t_ (mL/min/kg)	0.92	Ramael et al. (2006)
Cl_r_ (mL/min/kg)	0.63	Patsalos (2000)
GFR^a^ fup (mL/s)	0.8	Calculated
Pediatric (4–12 years)		
*V*_ss_ (L)	10.39	Calculated, (Poulin and Theil, 2002)
Cl_t_ ped (mL/min/kg)	1.43	Fountain et al. (2007)
Cl_r_ ped (mL/min/kg)	0.95	Calculated
GFR^a^ fup ped (mL/s)	0.52	Calculated
Pediatric (0.5–3 years)		
*V*_ss_ (L)	6.04	Calculated, Poulin and Theil (2002)
Cl_t_ ped (mL/min/kg)	1.46	Glauser et al. (2007)
Cl_r_ ped (mL/min/kg)	1.12	Calculated
GFR^a^ fup ped (mLs)	0.30	Calculated

^a^ Drugbank accessed by https://www.drugbank.ca/drugs/DB01202: MW: molecular weight; logP: log of the octanol-water partition coefficient for the neutral compound; pKa: acid dissociation constant; Fup: fraction unbound; V_ss_: volume of distribution at steady state; B/P: blood/plasma ratio concentration, Fabs: absorbed fraction; Kp: tissue: plasma partition coefficient; Cl_t_: total clearance from intravenous; Cl_r_: renal clearance; GFR: glomerular filtration rate; ped: pediatrics.

Pharmacokinetic data for LEV, encompassing intravenous and oral administration as well as single and multiple-dose regimens, were collected from the literature for model development and qualification. Only studies that provided clear information on age, sex, height, weight, renal function, dosing, and plasma concentration profiles were included (Supplementary Table S1). Plasma concentration-time profiles were digitally extracted using WebPlotDigitizer 4.2 (Rohatgi, 2021) to enable overlay with model-predicted profiles. For publications lacking reported summary exposure parameters, the area under the curve (AUC) and the maximum concentration (C_max_) were estimated from the average profiles using non-compartmental analysis.

A complete clinical dataset from a previous published study including Brazilian population (Freitas-Lima et al., 2011) was used to support model validation. All subjects gave their written consent, and the protocol was approved by the Ethics Committee of Hospital das Clínicas, Ribeirão Preto School of Medicine, Brazil (Freitas-Lima et al., 2011). This dataset included 15 adult patients with epilepsy (8 males and 7 females; age range: 19–51 years, mean age: 39) who were admitted to the Clinical Research Unit of the Clinical Hospital of Ribeirão Preto Medical School. Pregnant women or those at risk for pregnancy were excluded. Each patient received a single 1000 mg oral dose of levetiracetam (UCB, Italy) in a fasting state with 50 mL of water. Blood samples were collected at pre-dose and at 1, 3, 6-, 9-, 12-, and 24-h post-dose. Plasma LEV concentrations were measured as described in the original publication (Freitas-Lima et al., 2011).

### 2.2 Model performance verification

The exposure metrics predicted by the PBPK model were compared with *in vivo* concentration-time profiles after oral or intravenous administration collected from the literature. Virtual populations consisting of 100 subjects per simulation were generated to closely match the characteristics of participants in the corresponding clinical study. Each simulation used the same study design parameters, such as dose, route of administration, age, sex distribution, ethnicity (*e.g.,* White, Japanese or Chinese) and body weight, as reported in the respective studies (Supplementary Table S1).

The suitability of the PBPK model was assessed by comparing the predicted plasma concentration-time profile with the observed clinical data from each study. Model verification was deemed successful when key PK parameters, C_max_ and area AUC, were predicted within two-fold range of the corresponding observed values (Jones et al., 2009; Medicines Agency, 2018; Obach et al., 1997). To quantitatively assess model accuracy, the geometric mean fold-error (GMFE) was calculated for both AUC and C_max_ using Equation 1.

$$GMFE=10^{\frac{\sum \left|\log_{10}\left(\frac{pred\;PK\;parameter}{obs\;PK\;parameter}\right)\right|}{n}}$$ (1)

### 2.3 Pediatric PBPK model and dose scaling

The verified adult PBPK model was scaled to the pediatric population (infant and children) using the GastroPlus Population Estimates for Age-Related Physiology™ module. Extrapolation from adults to pediatrics was primarily based on differences in glomerular filtration rate (GFR) and unbound plasma protein fraction (*f*_*up*_). Plasma concentration profiles over time for pediatric patients aged 6 months to 12 years receiving doses ranging from 20 to 60 mg/kg were collected from the literature (Fountain et al., 2007; Glauser et al., 2002; Pellock JM et al., 2001) (Supplementary Material, Supplementary Table S1). The pediatric model was considered acceptable when the primary pharmacokinetic parameters (C_max_ and AUC) were predicted within a two-fold range of the corresponding observed values.

The qualified pediatric PBPK model was then used to simulate various doses, ranging from 10 to 120 mg/kg, administered either twice a day (BID) or three times a day (TID), across four predefined age groups: a) 0.5–3 years; b) 4–6 years; c) 7–9 years; and d) 10–12 years. These age categories were defined based on the availability of supporting clinical data to optimize age-appropriate dose recommendations. Simulations were performed at steady-state conditions (SS).

Dose suitability was evaluated using two PK targets: through concentration (C_tr_) and C_max_ at the SS. The plasma therapeutic window for adults assumed was between 5 and 46 mg/L which was established based on previous studies (Patsalos et al., 2008; Reimers et al., 2018; Stepanova and Beran, 2014). The efficacy target was defined as achieving a steady-state C_tr_ above the lower bound of 5 mg/L and the safety target was defined as maintaining a steady-state C_max_ below the upper bound of 46 mg/L. Population simulations were performed as 10 repeated trials, each with 15 virtual pediatric subjects per age group. All graphs were generated using R version 4.4.1, with the ggplot2 package (v. 3.5.1).

### 2.4 Probability of target attainment (PTA)

The dataset containing the anthropometric characteristics (age, weight, height and sex), dose, C_max_ and Ctr at the SS for all simulated pediatric individual, according to Section 2.3, were assumed to evaluate the PTA for pediatric population.

The efficacy target was a C_tr_ higher than 5 mg/L, and for the safety target was a C_max_ lower than 46 mg/L. The PTA was calculated using R version 4.4.1.

### 2.5 Multivariate linear regression analysis to guide the first dose prescription

From the simulated pediatric trial dataset, variables for each dose, dosing regimen and age group were used as data to construct the multivariate linear regression model. For each virtual patient subject, covariates such as age, weight, height, body surface area (BSA), GFR and daily dose (DD) were included, along with the pharmacokinetic outcomes, C_max_ and C_tr_ at SS. C_max_ or C_tr_ were set as dependent variables, and all other covariates were assessed as potential predictors. A forward stepwise inclusion was employed, with each included factor evaluated for statistical significance (p < 0.05) and multicollinearity. The regression analysis was conducted using the tidyverse package in Rstudio.

The multivariate linear equation has the general model as presented in Equation 2.

$$DV=\beta_{0}+\left(\beta_{1}*x_{1}\right)+\left(\beta_{2}*x_{2}\right)+\left(\beta_{3}*x_{3}\right)+\left(\beta_{4}*x_{4}\right)+\ldots +\left(\beta_{n}*x_{n}\right)+\varepsilon$$ (2)

where: β represents each coefficient related to each factor x and ɛ is the standard error of regression.

## 3 Results

### 3.1 Adult PBPK model

**FIGURE 1:**
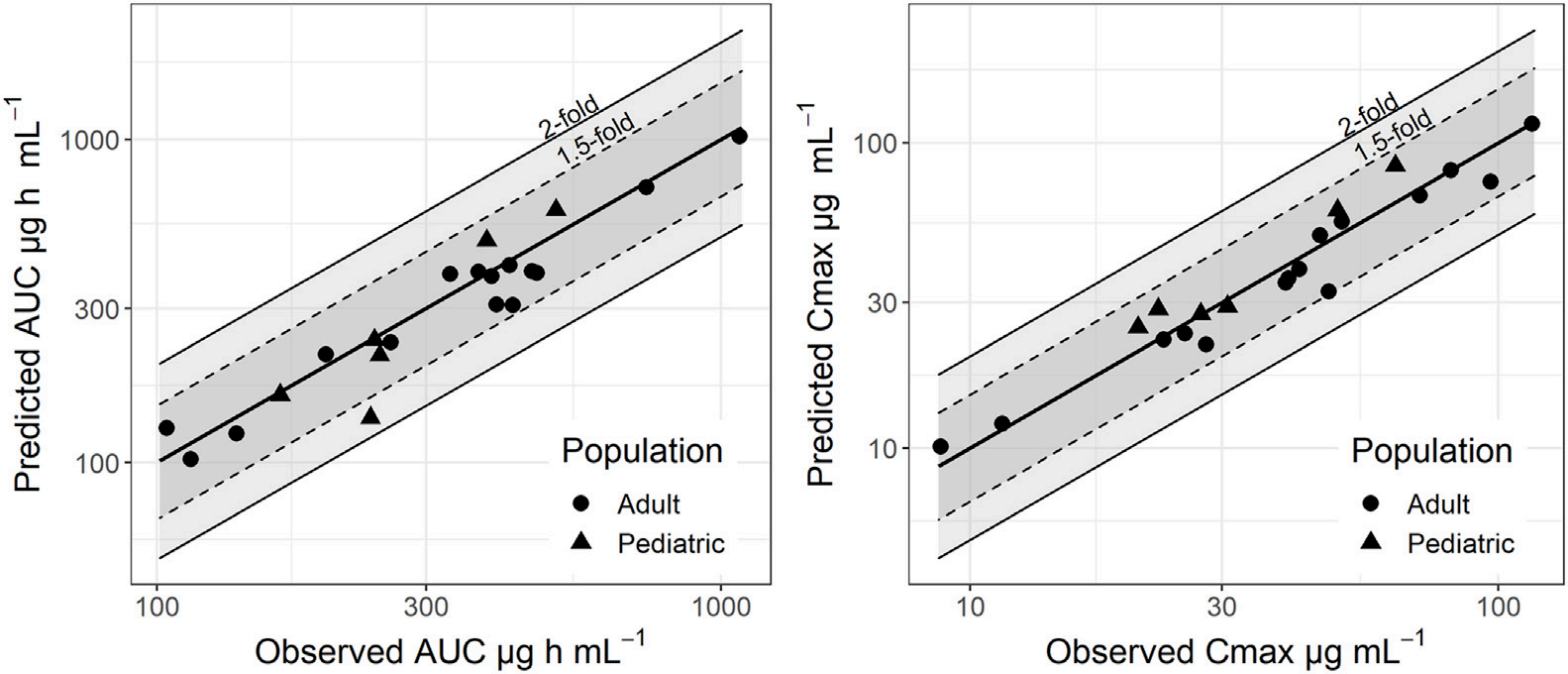
Predictive performance plots for the levetiracetam PBPK model comparing predicted and observed AUC and Cmax. Symbols represent the performance for adults (circle) and pediatrics from 6 months to 12 years old (triangles). Solid bold line represents the line of unity, dashed lines and solid lines represent the 2.0-fold and 1.5-fold difference, respectively.

A PBPK model for levetiracetam was developed and verified in healthy adult volunteers after oral or intravenous administration of single/multiple doses at various dose levels. The model was able to predict AUC and C_max_ within a two-fold range of observed clinical data used for model development and verification (Figure 1). Predicted-to-observed ratios for both intravenous and oral dosing scenarios are provided in the Supplementary Table S2. The model was considered verified and used as the basis for extrapolation to the pediatrics population.

### 3.2 Dose scaling by PBPK model and probability of target attainment

**FIGURE 2:**
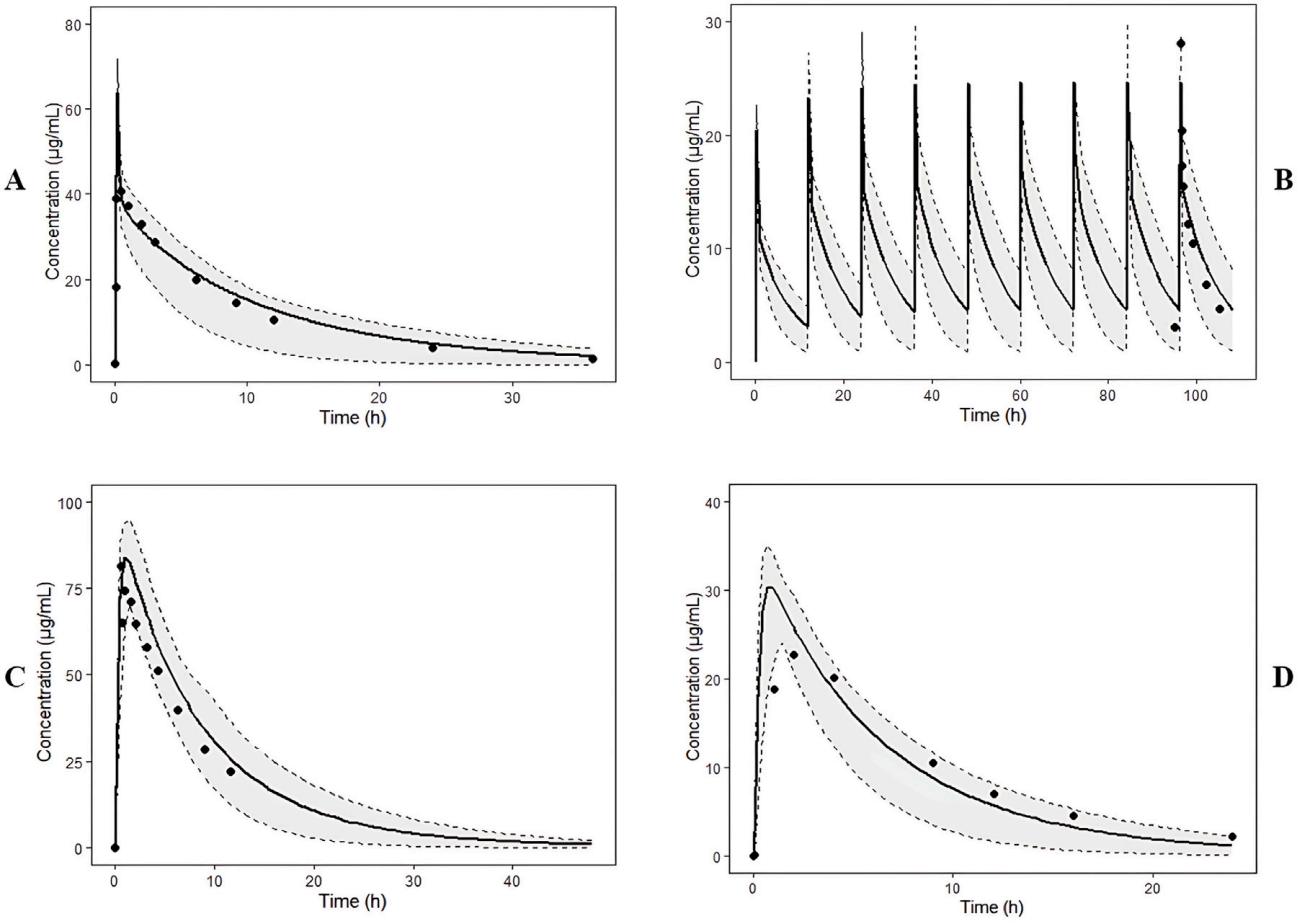
Predicted and observed plasma concentration profiles of LEV. The solid lines represent mean simulated, grey areas between dashed lines represent 5-95th percentiles of predictions and the black circles are observed data from each experimental condition. The observed data were from **(A)** adult healthy volunteers after a single 15-min IV infusion of 1500 mg (Ramael et al., 2006), **(B)** adult healthy volunteers after multiple IV administration of 500 mg (Spencer et al., 2011), **(C)** adult healthy volunteers after a single oral administration of 1000 mg (Freitas-Lima et al., 2011), **(D)** pediatric patients (6–12 years old) after a single oral administration of 20 mg kg^-1^ (Pellock et al., 2001).

The adult PBPK model was successfully extrapolated to the pediatric population (0.5–12 years old). Model performance was verified against published clinical data, with predicted AUC and Cmax values falling within a 2-fold margin of observed data (Figures 1, 2; Supplementary Table S2; Table S3).

To guide dose optimization, pharmacokinetic targets were defined based on established clinical evidence. Efficacy is primarily associated with maintaining a sufficient trough concentration (C_tr_), while safety is related to avoiding excessively high peak concentrations (C_max_).

Simulations revealed a challenge in achieving the efficacy target with standard dosing. To achieve a C_tr_ > 5 mg/L, our model indicates that high daily doses are required often exceeding 90 mg/kg for children up to 9 years old and 80 mg/kg for those 10–12 years old. The choice of dosing regimen twice-daily (BID) versus three-times-daily (TID) was critical in balancing efficacy and safety. For BID regimen while lower doses (*e.g.,* 40–70 mg/kg/day) can keep the C_max_ within the safety limit, the corresponding C_tr_ frequently falls below the 5 mg/L efficacy threshold. On the other hand, a TID regimen allows for higher total daily doses required for efficacy while mitigating the risk of high peak concentrations. For example, a DD of 90 mg/kg administered TID can achieve the therapeutic trough while keeping the C_max_ manageable.

**FIGURE 3:**
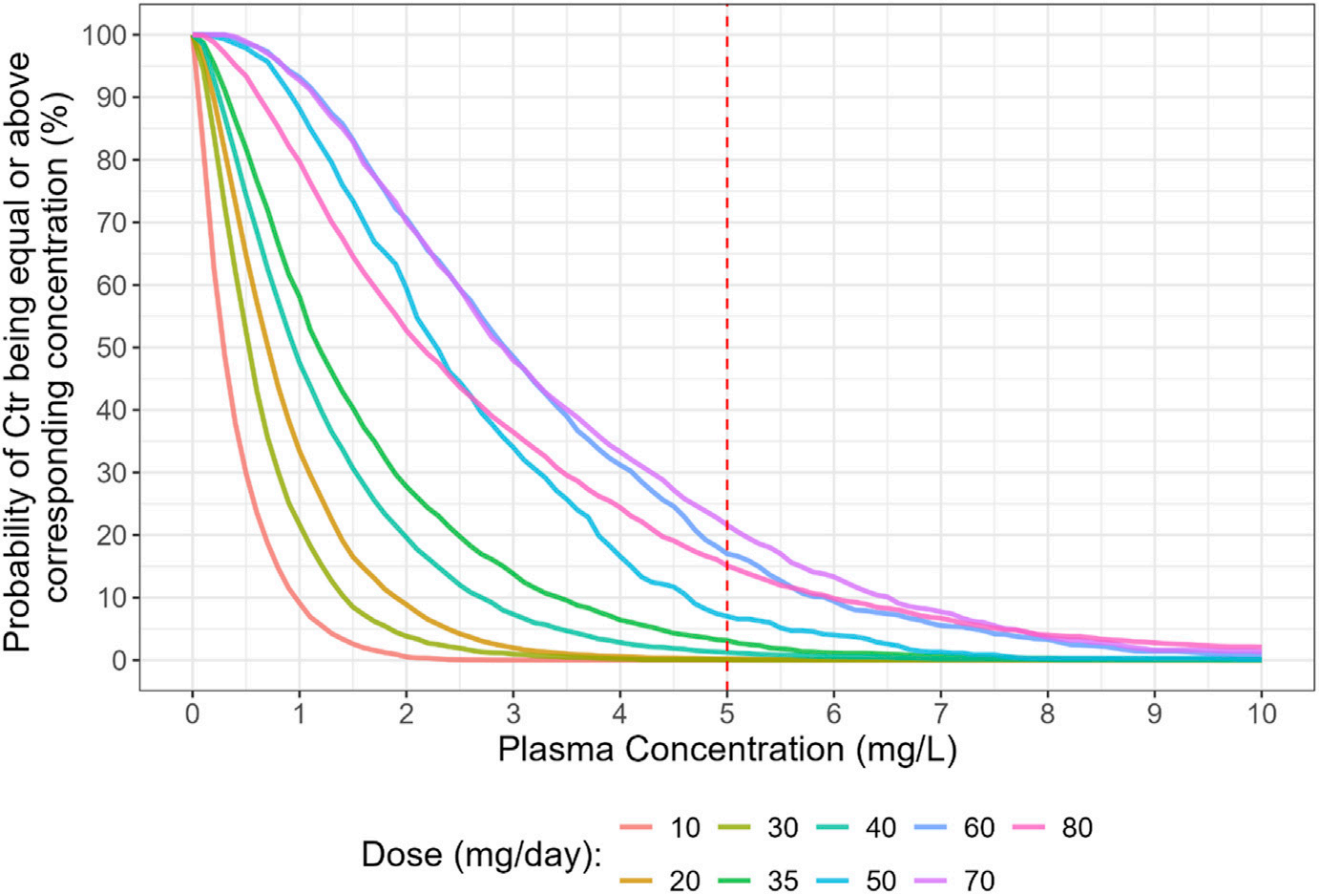
Probability of target attainment (PTA) for trough concentration (C_tr_), considering the plasma concentration range between 5 and 46 mg/mL. Daily Dose tested were 10, 20, 30, 35, 40, 50, 60, 70 and 80 mg/day.

**FIGURE 4:**
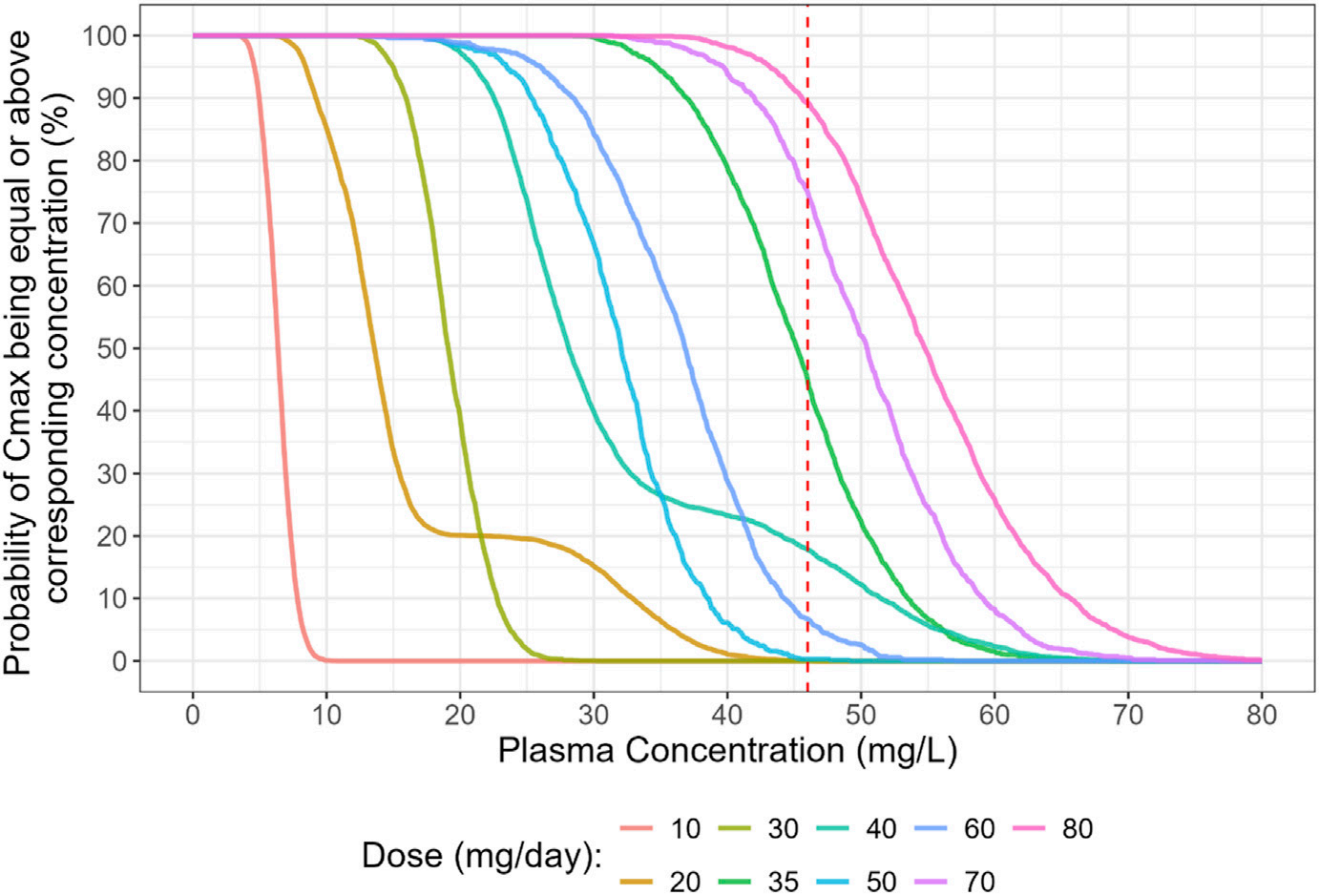
Probability of target attainment (PTA) for C_max_, considering the plasma concentration above 100 mg/mL. Daily Dose tested were 10, 20, 30, 35, 40, 50, 60, 70 and 80 mg/day.

The Probability of Target Attainment (PTA) analysis confirms this finding (Figures 3, 4). For the BID regimen, the probability of achieving a C_tr_ > 5 mg/L was low across the tested dose ranges. The TID regimen, however, showed a markedly improved PTA, making it the superior strategy for reliably achieving therapeutic goals in the pediatric population.

Finally, these weight-based recommendations are most relevant for children weighing less than 50 kg. For heavier children and adolescents, total daily doses should align with adult recommendations (1000–3000 mg/day) to achieve the desired therapeutic target.

### 3.3 Multivariate linear regression analysis to guide the first dose prescription

The multivariate linear regression results are presented in Table 2. Only statistically significant variables were kept in the final model, after assessing inter-variable correlations. The significant independent variables were dose, weight, dosing regimen and GFR. When C_tr_ was the dependent variable, the adjusted R2 indicated that the model explained 72% of the variability in the data. In contrast, when C_max_ was the dependent variable, the adjusted R^2^ demonstrated that the model explained about 94% of the variability. Considering the better predictive performance for C_max_ and greater clinical relevance supported by previous studies, only the C_max_-based model was considered.

**TABLE 2:** Multivariate linear regression results for C_max_ and C_trough_ as dependent variable.

Dependent variable C_tr_	
Analysis	Results
R^2^	0.7226
R^2^ Adjusted	0.7207
Standard error	1.521
Degrees of freedom	565
p-value	<2 E−16

ANOVA					
Source	DF	Sum Sq	Mean Sq	F value	p-value
Weight	1	587.56	587.56	253.92	<2.2 E−16
Dose	1	2098.80	2098.80	907.00	<2.2 E−16
Regimen	1	314.63	314.63	135.97	<2.2 E−16
GFR	1	405.26	405.26	175.13	<2.2 E−16
Residual	565	1307.42	2.31		

Parameter	Coefficients	Standard error	p-value
Intersection (β_0_)	−3.2426	0.2838	<2.2 E−16
Dose (β_1_)	0.0517	0.0020	<2.2 E−16
Weight (β_2_)	0.1633	0.0080	<2.2 E−16
Regimen (β_3_) 1 for BID and 2 for TID	1.4815	1.4815	<2.2 E−16
GFR	−2.9866	0.2257	<2.2 E−16

Dependent Variable Cmax	
Analysis	Results
R^2^	0.9390
R^2^ Adjusted	0.9385
Standard error	4.205
Degrees of freedom	565
p-value	<2.2 E−16

ANOVA					
Source	DF	Sum Sq	Mean Sq	F value	p-value
Weight	1	5149	5149	291.15	<2.2 E−16
Dose	1	139513	139513	7888.95	<2.2 E−16
Regimen	1	7666	7666	433.51	<2.2 E−16
GFR	1	1381	1381	78.08	<2.2 E−16
Residual	565	9992	18		

Parameter	Coefficients	Standard error	p-value
Intersection (β_0_)	11.9225	0.78469	<2.2 E−16
Dose (β_1_)	0.5034	0.0055	<2.2 E−16
Weight (β_2_)	0.2865	0.0222	<2.2 E−16
Regimen (β_3_) 1 for BID and 2 for TID	−7.8278	0.3696	<2.2 E−16
GFR	−5.5128	0.6239	<2.2 E−16

Ctr, Trough concentration; Cmax maximum concentration; R^2^: R-squared, the coefficient of determination; R^2^ Adjusted, Adjusted R-squared; ANOVA, analysis of variance; DF, degrees of freedom; Sum Sq, Sum of Squares; Mean Sq, Mean Square; F value, F-statistic; GFR, glomerular filtration rate; β0, The intercept of the regression model; β1,β2,β3, The regression coefficients for the variables Dose, Weight, and Regimen; BID, twice a day administration; TID, three times a day administration.

The multivariate linear equation has the general model as presented in Equation 2 and the results show the significant regression values for both regimen and the final model for pediatric dose suggestion for the first prescription is presented as Equations 3, 4.

$$C_{\max_{SS}}=\left[11.92\pm 0.79\right]+\left[\left(0.50\pm 0.29\right)*Dose\right]+\left[\left(0.28\pm 0.02\right)*Weight\right]-\left[\left(7.82\pm 0.37\right)*Reg\right]-\left[\left(5.51\pm 0.62\right)*GFR\right]$$ (3)

where: C_maxSS_ is given as mg. L^-1^; dose is given as mg.kg^-1^.day^-1^, WT is weight in kg, and Reg is the Regimen as 1 for BID and 2 for TID and GFR is the glomerular filtration rate in mL.sec^-1^.

When adjustment errors are removed from Equation 3, it could be simplified as Equation 4.

$$C_{\max_{SS}}=\left(11.92\right)+\left(0.50*Dose\right)+\left(0.28*Weight\right)-\left(7.82*Reg\right)-\left(5.51*GFR\right)$$ (4)

However, for the clinical routine, only the C_tr_ is possible to access. Then, the prescriber can define the desirable C_max_ by Equation 4 and estimate the C_tr_ at SS by Equation 5.

$$C_{{tr}_{SS}}=C_{\max_{SS}}*e^{-k_{e}*\left(\tau -1\right)}$$ (5)

where*:* $C_{{tr}_{SS}}$ *is the predicted minimal concentration at Steady-State when the* $C_{\max_{SS}}$ *(predicted maximum concentration at the Steady-State by* Equation 4 *) is assumed; k*_*e*_ *is the elimination constant, experimentally determined for each individual and τ (tau) is the dosing interval (hour). This relationship allows a clinician to estimate a patient’s individual elimination rate constant (ke) by using a clinically measured trough level* $C_{{tr}_{SS}}$ *, which can then inform subsequent dose adjustments.*

## 4 Discussion

LEV is considered one of the safest antiepileptic drugs, with its primary elimination via glomerular filtration being a key determinant of its favorable safety profile (Patsalos, 2000). In pediatric patients, LEV is often preferred due to its established efficacy, safety, linear pharmacokinetics, and minimal drug-drug interactions (Glauser et al., 2002). Despite its widespread use, optimizing initial dose selection in children remains a challenge. Therefore, this study aimed to develop and qualify a PBPK model to provide a more rational basis for initial dose prescription of levetiracetam in the pediatric population.

The development process began by building a whole-body PBPK model for healthy adults, which was successfully qualified against clinical data from Caucasian, Japanese, and Chinese populations. The model predicted the pharmacokinetic profile of LEV in adults to within a 2-fold error margin of observed *in vivo* data, establishing a reliable base model for pediatric extrapolation.

Subsequently, the adult model was scaled to a pediatric population (6 months–12 years old). This extrapolation was mechanistically anchored to age-dependent changes in glomerular filtration rate and the unbound plasma protein fraction, a methodology consistent with established pediatric modeling practices (Zhang et al., 2019). Our model incorporated a physiologically scaled GFR, which is dependent on organ size and blood flow. A key refinement was the reduction of the standard GFR value (approx. 120 mL/min/1.73 m^2^) to account for the known partial tubular reabsorption of LEV (Patsalos, 2000). This adjustment, applied proportionally across all pediatric age groups, was crucial for accurately capturing the drug elimination process. This approach is supported by recent PBPK work from Maglalang et al. (2024), who also noted that LEV renal clearance is substantially lower than the GFR, confirming significant tubular reabsorption (Germovsek et al., 2019; Zhang et al., 2019; Maglalang et al., 2024). As demonstrated in the results, the final model predictive accuracy was critically dependent on this GFR adjustment, thereby verifying our approach (see Supplementary Table S2; Figure 1).

The pediatric PBPK model was able to predict the pharmacokinetic profile of levetiracetam across all ages tested in this study, with a GMFE range of 0.77–1.32. Analysis of the virtual pediatric population showed that children weighing over 50 kg achieved C_max_ comparable to adults when receiving the same dose, validating the current weight-based cutoff for adult dosing. The model was then used to explore the safety and efficacy of alternative dosing strategies.

**FIGURE 5:**
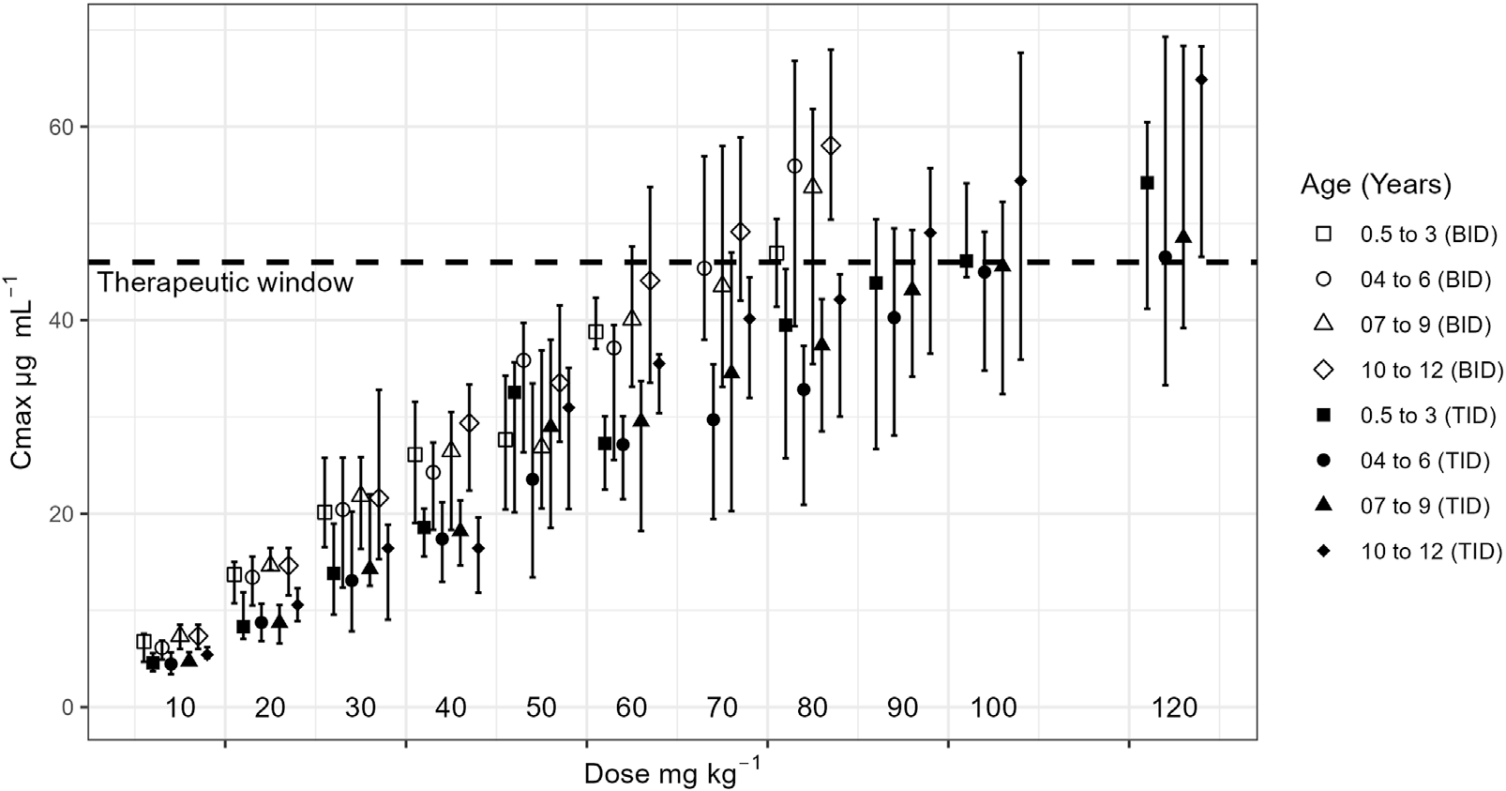
Maximum plasma concentration (C_max_) for each dose by age groups for a BID and TID regimen. Dashed lines represent the therapeutic window.

Critically, the model identified the TID regimen as a safer strategy for dose escalation in younger children (0.5–9 years). As shown in Figure 5, this regimen allows for the daily dose to be increased up to 90 mg/kg while maintaining C_max_ below the 46 mg/L safety boundary. This finding provides a mechanistic basis for the successful use of high doses (90–120 mg/kg/day) reported in clinical studies by Depositario-Cabacar et al. (2010) and Mandelbaum et al. (2005), where seizure frequency was reduced without significant safety concerns. It also aligns with a case report of a 7-year-old who tolerated 200 mg/kg/day without adverse effects (Kartal, 2017). The crucial clinical message from our findings is that for children aged 0.5–9 years who are not responding sufficiently to standard doses, clinicians could more confidently and safely escalate the dose up to 90 mg/kg/day by switching to a TID regimen. This approach mitigates plasma fluctuations and reduces the risk of peak-concentration-related side effects, allowing for a wider therapeutic dose range than is currently recommended (UCB, Biosciences, 1999) (Depositario-Cabacar et al., 2010).

To further enhance clinical utility, the mechanistic simulation results from the PBPK model were leveraged to develop a simple statistical tool. While traditional population pharmacokinetic (PopPK) modeling is a standard method for creating covariate-based dosing equations from clinical data, our PBPK-generated dataset was better suited for a different purpose. The PBPK model served as the mechanistic engine to generate a rich dataset, and a multivariate linear regression (MLR) model was then applied as a pragmatic ‘translator’ to distill these complex outputs into a simple algebraic formula for clinical use. This statistical approach successfully correlated C_max_ with key predictors identified by the PBPK model (body weight, dose, dosing regimen, and GFR), which together explained 94% of the variance in the simulated data. The resulting mathematical correlation (Equation 4) offers a practical tool for estimating an appropriate starting dose for pediatric patients (>0.5 years, <50 kg) on LEV monotherapy. This data-driven approach supports a more aggressive dose initiation strategy; if a concentration within the therapeutic range in desired, the initial titration steps could potentially be bypassed in favor of a starting dose predicted to directly achieve the therapeutic target, accelerating the time to clinical response.

This work compares favorably with other recent PBPK models for pediatric LEV. While our study focused on dose regimen optimization across a broad pediatric age range (0.5–12 years), the model by Maglalang et al. (2024) focused specifically on the effects of obesity and maturation in children from 1 month to 19 years. Their finding that obesity can alter weight-normalized clearance complements our work, suggesting that patient-specific factors like body composition are important considerations. Both studies underscore the central role of renal function in LEV disposition and validate the utility of PBPK modeling in refining pediatric dosage.

The limitations of this study must be acknowledged. The PBPK model was developed for LEV monotherapy in patients without significant comorbidities, whereas clinical practice often involves polytherapy. Potential interactions with other antiepileptic drugs (May et al., 2003; Florek-Luszczki et al., 2014) were not considered. Similarly, as our primary focus was on the maturational effects on clearance, the model was validated in populations with normal renal function, and its performance in patients with renal impairment has not been evaluated. Validating the model against data from subjects with varying degrees of renal impairment would be a valuable future step to further confirm the model mechanistic handling of renal clearance and expand its domain of applicability. Furthermore, our model simplifies renal elimination by empirically scaling the physiological glomerular filtration rate (GFR) downwards to match observed renal clearance, thereby implicitly accounting for tubular reabsorption. While the specific transporters involved in LEV reabsorption are not yet fully elucidated, future PBPK models could incorporate mechanistic transport kinetics (*e.g.,* for Organic Anion Transporters (OAT) or other transporters) as this information becomes available, which would further refine predictive accuracy.

Although the PBPK-based MLR equation provides a simple and powerful tool for clinicians, its own limitations must be understood. The MLR model is a statistical abstraction of the PBPK model’s output, not a mechanistic model itself. Its predictive accuracy is therefore entirely dependent on the validity of the underlying PBPK simulations. Furthermore, its use is restricted to the range of covariates (e.g., age, weight, GFR) evaluated in this study and cannot be extrapolated. As such, it requires validation with new, independent pediatric clinical data before it can be recommended for routine clinical use.

## 5 Conclusion

In conclusion, the PBPK model developed for levetiracetam in adults and pediatric patients (6 months–12 years old) was successfully verified and accurately predicted the drug pharmacokinetic profile. The pediatric model was subsequently used to explore the safe administration of doses exceeding currently recommended limits. The study demonstrated that therapeutic goals could be achieved with doses between 30 and 60 mg/kg/day using a BID regimen and showed that doses could be safely escalated up to 90 mg/kg/day in younger children by employing a TID regimen. To facilitate clinical translation, a multivariate linear regression model was developed that effectively predicted C_max_ based on body weight, dose, regimen, and glomerular filtration rate. The robustness of this framework is founded upon the core PBPK model key methodological correction for renal tubular reabsorption. While these findings provide a more rational approach to LEV dosing, prospective clinical validation is essential to confirm the model predictions and ensure its applicability across diverse clinical scenarios. It is also important to note that our model was developed using data from immediate-release (IR) oral formulations; therefore, this framework is specific to IR levetiracetam and would require re-parameterization to be applied to other formulations, such as extended-release.

## Data Availability

The original contributions presented in the study are included in the article/Supplementary Material, further inquiries can be directed to the corresponding author.

## Supplementary material

SUPPLEMENTARY TABLE S3 Simulated mean maximum concentration (Cmax), steady-state plasma concentration range, and their corresponding coefficient of variation (CV%) in the pediatric population

## References

[B21] Boberg M. Vrana M. Mehrotra A. Pearce R. E. Gaedigk A. Bhatt D. K. (2017). Age-dependent absolute abundance of hepatic carboxylesterases (CES1 and CES2) by LC-MS/MS proteomics: application to PBPK modeling of oseltamivir *in vivo* pharmacokinetics in infants. Drug Metab. Dispos. 45 (2), 216–223. 10.1124/dmd.116.072652 27895113 PMC5267516

[B1] Celdran de Castro A. Nascimento F. A. Beltran-Corbellini Á. Toledano R. Garcia-Morales I. Gil-Nagel A. (2023). Levetiracetam, from broad-spectrum use to precision prescription: a narrative review and expert opinion. Seizure Eur. J. Epilepsy 107, 121–131. 10.1016/j.seizure.2023.03.017 37023625

[B2] Depositario‐Cabacar D. T. Peters J. M. Pong A. W. Roth J. Rotenberg A. Riviello J. J. (2010). High‐dose intravenous levetiracetam for acute seizure exacerbation in children with intractable epilepsy. Epilepsia 51 (7), 1319–1322. 10.1111/j.1528-1167.2010.02519.x 20163437

[B22] Di L. (2019). The impact of carboxylesterases in drug metabolism and pharmacokinetics. Curr. Drug Metab. 20 (2), 91–102. 10.2174/1389200219666180821094502 30129408 PMC6635651

[B3] Drugbank. Levetiracetam (2021). Available online at: https://go.drugbank.com/drugs/db01202.

[B23] Florek-Luszczki M. Wlaz A. Luszczki J. J. (2014). Interactions of levetiracetam with carbamazepine, phenytoin, topiramate and vigabatrin in the mouse 6Hz psychomotor seizure model - a type II isobolographic analysis. Eur. J. Pharmacol. 723, 410–418. 10.1016/j.ejphar.2013.10.063 24211788

[B24] Franco V. Canevini M. P. Capovilla G. De Sarro G. Galimberti C. A. Gatti G. (2014). Off-label prescribing of antiepileptic drugs in pharmacoresistant epilepsy: a cross-sectional drug utilization study of tertiary care centers in Italy. CNS Drugs 28 (10), 939–949. 10.1007/s40263-014-0189-8 25056568

[B4] Fountain N. B. Conry J. A. Rodríguez-Leyva I. Gutierrez-Moctezuma J. Salas E. Coupez R. (2007). Prospective assessment of levetiracetam pharmacokinetics during dose escalation in 4- to 12-year-old children with partial-onset seizures on concomitant carbamazepine or valproate. Epilepsy Res. 74 (1), 60–69. (Sarah). 10.1016/j.eplepsyres.2006.12.005 17270398

[B5] Freitas-Lima P. Alexandre V. Pereira L. R. L. Feletti F. Perucca E. Sakamoto A. C. (2011). Influence of enzyme inducing antiepileptic drugs on the pharmacokinetics of levetiracetam in patients with epilepsy. Epilepsy Res. 94 (1–2), 117–120. 10.1016/j.eplepsyres.2011.01.007 21282041

[B25] Germovsek E. Barker C. I. S. Sharland M. Standing J. F. (2019). Pharmacokinetic-pharmacodynamic modeling in pediatric drug development, and the importance of standardized scaling of clearance. Clin. Pharmacokinet. 58(1):39–39152. 10.1007/s40262-018-0659-0 29675639 PMC6325987

[B6] Glauser T. A. Pellock J. M. Bebin E. M. Fountain N. B. Ritter F. J. Jensen C. M. (2002). Efficacy and safety of levetiracetam in children with partial seizures: an open-label trial. Epilepsia 43 (5), 518–524. 10.1046/j.1528-1157.2002.13101.x 12027913

[B7] Glauser T. A. Mitchell W. G. Weinstock A. Bebin M. Chen D. Coupez R. (2007). Pharmacokinetics of levetiracetam in infants and young children with epilepsy. Epilepsia 48 (6), 1117–1122. 10.1111/j.1528-1167.2007.01090.x 17442002

[B9] Jones H. M. Gardner I. B. Watson K. J. (2009). Modelling and PBPK simulation in drug discovery. AAPS J. 11 (1), 155–166. 10.1208/s12248-009-9088-1 19280352 PMC2664888

[B26] Maglalang P. D. Sinha J. Zimmerman K. McCann S. Edginton A. Hornik C. P. (2024). Application of physiologically based pharmacokinetic modeling to characterize the effects of age and obesity on the disposition of levetiracetam in the pediatric population. Clin. Pharmacokinet. 63 (6), 885–899. 10.1007/s40262-024-01367-2 38814425 PMC11225543

[B10] Mangelings D. Saevels J. Vander Heyden Y. (2006). Enantiomeric impurity determination of levetiracetam using capillary electrochromatography. J. Sep. Sci. 29 (18), 2827–2836. 10.1002/jssc.200600190 17305245

[B11] Masungi C. Mensch J. Van Dijck A. Borremans C. Willems B. Mackie C. (2008). Parallel artificial membrane permeability assay (PAMPA) combined with a 10-day multiscreen Caco-2 cell culture as a tool for assessing new drug candidates. Pharmazie 63 (3), 194–199. 10.1691/ph.2008.7327 18444507

[B27] May T. W. Rambeck B. Jürgens U. (2003). Serum concentrations of levetiracetam in epileptic patients: the influence of dose and co-medication. Ther. Drug Monit. 25 (6), 690–699. 10.1097/00007691-200312000-00007 14639055

[B12] Medicines Agency E. (2018). Committee for medicinal products for human use (CHMP) guideline on the reporting of physiologically based pharmacokinetic (PBPK) modelling and simulation. Available online at: www.ema.europa.eu/contact.

[B13] Obach R. S. Baxter J. G. Liston T. E. Silber B. M. Jones B. C. MacIntyre F. (1997). The prediction of human pharmacokinetic parameters from preclinical and *in vitro* metabolism data. J. Pharmacol. Exp. Ther. 283 (1), 46–58. 10.1016/s0022-3565(24)36999-x 9336307

[B14] Patsalos P. N. (2000). Pharmacokinetic profile of levetiracetamtoward ideal characteristics. Pharmacol. Ther. 85 (2), 77–85. 10.1016/S0163-7258(99)00052-2 10722121

[B15] Patsalos P. N. Berry D. J. Bourgeois B. F. D. Cloyd J. C. Glauser T. A. Johannessen S. I. (2008). Antiepileptic drugs—best practice guidelines for therapeutic drug monitoring: a position paper by the subcommission on therapeutic drug monitoring, ILAE commission on therapeutic strategies. Epilepsia 49 (7), 1239–1276. 10.1111/j.1528-1167.2008.01561.x 18397299

[B16] Pellock J. M. Glauser T. A. Bebin E. M. Fountain N. B. Ritter F. J. Coupez R. M. (2001). Pharmacokinetic study of levetiracetam in children. Epilepsia 42 (12), 1574–1579. 10.1046/j.1528-1157.2001.41300.x 11879369

[B17] Petruševska M. Berglez S. Krisch I. Legen I. Megušar K. Peternel L. (2015). Biowaiver monographs for immediate release solid oral dosage forms: Levetiracetam. J. Pharm. Sci. 104 (9), 2676–2687. 10.1002/jps.24350 25663270

[B18] Poulin P. Theil F. P. (2002). Prediction of pharmacokinetics prior to *in vivo* studies. II. Generic physiologically based pharmacokinetic models of drug disposition. J. Pharm. Sci. 91 (5), 1358–1370. 10.1002/jps.10128 11977112

[B19] Ramael S. de Smedt F. Toublanc N. Otoul C. Boulanger P. Riethuisen J.-M. (2006). Single-dose bioavailability of levetiracetam intravenous infusion relative to oral tablets and multiple-dose pharmacokinetics and tolerability of levetiracetam intravenous infusion compared with placebo in healthy subjects. Clin. Ther. 28 (5), 734–744. 10.1016/j.clinthera.2006.05.004 16861095

[B28] Reimers A. Berg J. A. Burns M. L. Brodtkorb E. Johannessen S. I. Johannessen Landmark C. (2018). Reference ranges for antiepileptic drugs revisited: a practical approach to establish national guidelines. Drug Des. Devel Ther. 12, 271–280. 10.2147/DDDT.S154388 29467570 PMC5811172

[B29] Rohatgi A. (2021). WebPlotDigitiser. [Computer Software version 4.5] https://automeris.io/WebPlotDigitizer/ .

[B30] Shao W. Shen C. Wang W. Sun H. Wang X. Geng K. (2023). Development and validation of physiologically based pharmacokinetic model of levetiracetam to predict exposure and dose optimization in pediatrics. J. Pharm. Sci. 112 (10), 2667–2675. 10.1016/j.xphs.2023.03.025 37023853

[B31] Sinha J. Karatza E. Gonzalez D. (2022). Physiologically-based pharmacokinetic modeling of oxcarbazepine and levetiracetam during adjunctive antiepileptic therapy in children and adolescents. CPT Pharmacometrics Syst. Pharmacol. 11 (2), 225–239. 10.1002/psp4.12750 34816634 PMC8846633

[B32] Stepanova D. Beran R. G. (2014). Measurement of levetiracetam drug levels to assist with seizure control and monitoring of drug interactions with other anti-epileptic medications (AEMs). Seizure 23 (5), 371–376. 10.1016/j.seizure.2014.02.003 24630809

[B20] Strolin Benedetti M. Whomsley R. Nicolas J. M. Young C. Baltes E. (2003). Pharmacokinetics and metabolism of 14C-levetiracetam, a new antiepileptic agent, in healthy volunteers. Eur. J. Clin. Pharmacol. 59 (8–9), 621–630. 10.1007/s00228-003-0655-6 14530892

[B33] UCB Pharma, Inc. (1999). Keppra (Levetiracetam) prescribing information.

[B34] Wang D.-D. Jin Q. Hou J. Feng L. Li N. Li S.-Y. (2016). Highly sensitive and selective detection of human carboxylesterase 1 activity by liquid chromatography with fluorescence detection. J. Chromatogr. B 1008, 212–218. 10.1016/j.jchromb.2015.11.046 26673230

[B35] Zhang Y. Mehta N. Muhari-Stark E. Burckart G. J. van den Anker J. Wang J. (2019). Pediatric renal ontogeny and applications in drug development. J. Clin. Pharmacol. 59 (Suppl. 1), S9–S20. 10.1002/jcph.1490 31502684

