# Supplementary material for: Physiologically-based pharmacokinetic modeling and simulation for initial dose optimization of levetiracetam in pediatrics
Source: Front Pharmacol. 2025 Dec 4;16:1678960. doi: 10.3389/fphar.2025.1678960 (PMC12711802; doi:10.3389/fphar.2025.1678960)
Supplement: Supplementary file 1 [file Table1.docx]

Supplementary Material

# Supplementary Figures and Tables

Table S1- Summary of levetiracetam observed data available in literature used for model development and verification.

| **Dataset** | **Population** | **Age (years)** | **Dose (mg)** | **n** | **Route/ regimen** | **Reference** |
| --- | --- | --- | --- | --- | --- | --- |
| Datasets for model development | | | | | | |
| 1 | HV | 30-40 | 1500 | 17 | IV/SD | (Ramael et al., 2006) |
| 2 | HV | 18-50 | 3500 | 8 | Oral/SD | (Patsalos, 2000) |
| Datasets for model verification | | | | | | |
| 3 | HV | 19-51 | 1000 | 15 | Oral/SD | * |
| 4 | HV | 30-40 | 1500 | 17 | Oral/SD | (Ramael et al., 2006) |
| 5 | HV | 30-40 | 1500 | 12 | IV/MD | (Ramael et al., 2006) |
| 6 | HV | 40-54 | 500 | 12 | IV/MD | (Spencer et al., 2011) |
| 7 | HV | 18-50 | 500 | 8 | Oral/SD | (Patsalos, 2000) |
| 8 | HV | 18-50 | 1000 | 8 | Oral/SD | (Patsalos, 2000) |
| 9 | HV | 18-50 | 2000 | 8 | Oral/SD | (Patsalos, 2000) |
| 10 | HV | 18-50 | 5000 | 8 | Oral/SD | (Patsalos, 2000) |
| 11 | HV | 22-52 | 1500 | 16 | Oral/SD | (Coupez et al., 2003) |
| 12 | HVjap | 20-40 | 1500 | 26 | IV/SD | (Toublanc et al., 2015) |
| 13 | HVjap | 20-40 | 1500 | 26 | PO/SD | (Toublanc et al., 2015) |
| 14 | HVchi | 20-36 | 500 | 26 | PO/SD | (Zhao et al., 2007) |
| 15 | HVchi | 20-36 | 1500 | 26 | PO/SD | (Zhao et al., 2007) |
| 16 | PED | 06-12 | 20/kg | 24 | Oral/SD | (Pellock JM et al., 2001) |
| 17 | PED | 04-12 | 20/kg | 21 | Oral/SD | (Fountain et al., 2007) |
| 18 | PED | 04-12 | 40/kg | 21 | Oral/SD | (Fountain et al., 2007) |
| 19 | PED | 04-12 | 60/kg | 21 | Oral/SD | (Fountain et al., 2007) |
| 20 | PED | 0.5-02 | 20/kg | 6 | Oral/SD | (Glauser et al., 2007) |
| 21 | PED | 02-04 | 20/kg | 3 | Oral/SD | (Glauser et al., 2007) |

HV, Healthy Volunteers (HVjap, Japanese; HVchi, Chinese); PED, Pediatric patients; IV, Intravenous; PO, Oral; SD, Single Dose; MD, Multiple Dose. Doses presented as 'X/kg' indicate a dose of X mg per kg of body weight. For oral simulations, immediate-release (IR) oral formulations were used.

**Table S2**- Comparison of predicted and observed pharmacokinetic parameters (AUC, C_max_) of levetiracetam at different doses and regimen.

| **Dat.** | **Age** | **Dose** | **Pop.** | **OBS**  **AUC** | **PRED**  **AUC** | **Ratio** | **OBS**  **C_max_** | **PRED**  **C_max_** | **Ratio** | **Ref.** |
| --- | --- | --- | --- | --- | --- | --- | --- | --- | --- | --- |
|  |  | **mg** |  | **µg.h mL^-1^** | **µg.h mL^-1^** |  | **µg mL^-1^** | **µg mL^-1^** |  |  |
| 1 | 30-40 | 1500 | HV | 392.4 | 378.0 | 0.96 | 50.50 | 55.12 | 1.09 | (Ramael et al., 2006) |
| 2 | 18-50 | 3500 | HV | 737.1 | 711.8 | 0.97 | 81.34 | 81.17 | 1.00 | (Patsalos, 2000) |
| 3 | 19-51 | 1000 | HV | 259.85 | 236.11 | 0.91 | 25.5 | 23.70 | 0.93 | * |
| 4 | 30-40 | 1500 | HV | 427.9 | 307.0 | 0.71 | 47.70 | 32.51 | 0.68 | (Ramael et al., 2006) |
| 5 | 30-40 | 1500 | HV | 371.9 | 390.0 | 1.04 | 71.00 | 67.30 | 0.94 | (Ramael et al., 2006) |
| 6 | 40-54 | 500 | HV | 138.4 | 123.2 | 0.89 | 28.00 | 21.80 | 0.78 | (Spencer et al., 2011) |
| 7 | 18-50 | 500 | HV | 114.8 | 102.3 | 0.89 | 8.80 | 10.10 | 1.15 | (Patsalos, 2000) |
| 8 | 18-50 | 1000 | HV | 199.3 | 216.5 | 1.09 | 23.23 | 22.70 | 0.98 | (Patsalos, 2000) |
| 9 | 18-50 | 2000 | HV | 422.3 | 408.6 | 0.97 | 45.97 | 49.83 | 1.08 | (Patsalos, 2000) |
| 10 | 18-50 | 5000 | HV | 1077.9 | 1020.7 | 0.95 | 115.70 | 115.40 | 1.00 | (Patsalos, 2000) |
| 11 | 22-52 | 1500 | HV | 400.0 | 308.1 | 0.77 | 39.60 | 34.80 | 0.88 | (Coupez et al., 2003) |
| 12 | 20-40 | 1500 | HVjap | 462.09 | 390.8 | 0.84 | 96.70 | 74.60 | 0.77 | (Toublanc et al., 2015) |
| 13 | 20-40 | 1500 | HVjap | 471.95 | 385.9 | 0.81 | 41.94 | 38.55 | 0.91 | (Toublanc et al., 2015) |
| 14 | 20-36 | 500 | HVchi | 104.23 | 127.96 | 1.22 | 11.52 | 12.00 | 1.04 | (Zhao et al., 2007) |
| 15 | 20-36 | 1500 | HVchi | 331.04 | 383.89 | 1.15 | 40.06 | 36.00 | 0.89 | (Zhao et al., 2007) |
| 16 | 06-12 | 20/kg | PED | 248.2 | 214.9 | 0.87 | 22.7 | 28.5 | 1.26 | (Pellock et al., 2001) |
| 17 | 04-12 | 20/kg | PED | 165.5 | 162.1 | 0.98 | 20.8 | 24.83 | 1.19 | (Fountain et al., 2007) |
| 18 | 04-12 | 40/kg | PED | 384.3 | 486.2 | 1.27 | 49.6 | 60.15 | 1.21 | (Fountain et al., 2007) |
| 19 | 04-12 | 60/kg | PED | 510 | 604.5 | 1.19 | 63.80 | 84.09 | 1.32 | (Fountain et al., 2007) |
|  | **Table S2 - continuation** | | |  |  |  |  |  |  |  |
| **Dat.** | **Age** | **Dose** | **Pop.** | **OBS**  **AUC** | **PRED**  **AUC** | **Ratio AUC** | **OBS**  **C_max_** | **PRED**  **C_max_** | **Ratio C_max_** | **Ref.** |
|  |  | **mg** |  | **µg.h mL^-1^** | **µg.h mL^-1^** |  | **µg mL^-1^** | **µg mL^-1^** |  |  |
| 20 | 0.5-02 | 20/kg | PED | 239.0 | 137.76 | 0.57 | 27.32 | 27.46 | 0.99 | (Glauser et al., 2007) |
| 21 | 02-04 | 20/kg | PED | 242.8 | 239.6 | 1.01 | 30.69 | 29.12 | 1.05 | (Glauser et al., 2007) |

*Original data; **Dat**: dataset; **Pop**: Population; **HV**: Healthy adult volunteers; **HVjap**: Healthy adult volunteers, Japanese; **HVchi**: Healthy adult volunteers, Chinese; **PED**: Pediatric patients; **OBS**: Observed data; **PRED**: Predicted data; **AUC:** area under the curve; **C_max_:** maximum plasma concentration.

| **Age (Years)**  **[weight] (kg)** | **Dose**  **(mg kg^-1^ day^-1^)** | **BID** | | **TID** | | |
| --- | --- | --- | --- | --- | --- | --- |
|  |  | **C_max_**  **(µg mL^-1^)** | **C_th_**  **(µg mL^-1^)** | **C_max_**  **(µg mL^-1^)** | | **C_th_**  **(µg mL^-1^)** |
| **0.5 to 3**  **[8.29-14.95]** | **10** | 6.78 [13.24 %] | 0.31 | | 4.59 [11.62%] | 0.50 |
|  | **20** | 13.71 [9.67%] | 0.59 | | 9.33 [14.38%] | 1.14 |
|  | **30** | 20.15[9.39%] | 0.62 | | 13.82[14.33%] | 1.33 |
|  | **40** | 26.11[11.27%] | 0.78 | | 18.58 [9.32%] | 2.30 |
|  | **50** | 27.65[8.26%] | 1.32 | | 32.55[12.31%] | 2.18 |
|  | **60** | 38.81[32.03%] | 1.57 | | 27.27[13.14%] | 2.41 |
|  | **80** | 46.93[6.92%] | 2.74 | | 38.07[12.23%] | 4.63 |
|  | **90** |  |  | | 43.86[16.89%] | 4.82 |
|  | **100** |  |  | | 46.12[9.00%] | 5.22 |
|  | **120** |  |  | | 54.20[10.00%] | 6.16 |
| **4 to 6**  **[17.34-23.81]** | **10** | 6.15 [10.63 %] | 0.18 | 4.46 [13.57%] | | 0.42 |
|  | **20** | 13.43 [8.8%] | 0.94 | 8.75 [12.41%] | | 1.02 |
|  | **30** | 20.42[14.67%] | 0.64 | 13.08[8.75%] | | 1.17 |
|  | **40** | 24.27[11.93%] | 0.91 | 17.39[13.98%] | | 1.58 |
|  | **50** | 35.85[12.11%] | 0.81 | 23.55[10.94%] | | 2.40 |
|  | **60** | 37.14[10.57%] | 0.78 | 27.15[9.92%] | | 3.07 |
|  | **70** | 47.37[12.60%] | 0.98 | 29,72[10.83%] | | 3.15 |
|  | **80** | 55.94[11.11%] | 1.13 | 32.86[14.18%] | | 3.37 |
|  | **90** |  |  | 40.27[15.35%] | | 3.50 |
|  | **100** |  |  | 44.98[9.91%] | | 4.67 |
|  | **120** |  |  | 46.54[22.15%] | | 6.70 |
| **7 to 9**  **[26.54-34.45]** | **10** | 7.35[9.96%] | 0.98 | 4.74[14.46%] | | 0.32 |
|  | **20** | 14.63[11.56%] | 1.11 | 8.72[11.49%] | | 1.51 |
|  | **30** | 19.83[6.56%] | 1.14 | 14.29[11.03%] | | 2.00 |
|  | **40** | 26.46[14.98%] | 1.19 | 18.19[11.24%] | | 2.45 |
|  | **50** | 26.85[10.49%] | 1.83 | 28.96 [10.55] | | 2.70 |
|  | **60** | 40.07[9.52%] | 1.66 | 29.53[14.02%] | | 4.46 |
|  | **70** | 43.53[12.92%] | 1.98 | 34.52[15.72%] | | 4.59 |
|  | **80** | 53.75[13.29%] | 2.51 | 37.40[9.99%] | | 4.61 |
|  | **90** |  |  | 43.12[11.41%] | | 5.72 |
|  | **100** |  |  | 45.56[13.04%] | | 5.63 |
|  | **120** |  |  | 48.53[10.05%] | | 10.5 |

| **Age (Years)**  **[weight] (kg)** | **Dose**  **(mg kg^-1^ day^-1^)** | **BID** | | **TID** |  |
| --- | --- | --- | --- | --- | --- |
|  |  | **C_max_**  **(µg mL^-1^)** | **C_th_**  **(µg mL^-1^)** | **C_max_**  **(µg mL^-1^)** | **C_th_**  **(µg mL^-1^)** |
| **0.5 to 3**  **[8.29-14.95]** | **10** | 6.78 [13.24 %] | 0.31 | 4.59 [11.62%] | 0.50 |
|  | **20** | 13.71 [9.67%] | 0.59 | 9.33 [14.38%] | 1.14 |
|  | **30** | 20.15[9.39%] | 0.62 | 13.82[14.33%] | 1.33 |
|  | **40** | 26.11[11.27%] | 0.78 | 18.58 [9.32%] | 2.30 |
|  | **50** | 27.65[8.26%] | 1.32 | 32.55[12.31%] | 2.18 |
|  | **60** | 38.81[32.03%] | 1.57 | 27.27[13.14%] | 2.41 |
|  | **80** | 46.93[6.92%] | 2.74 | 38.07[12.23%] | 4.63 |
|  | **90** |  |  | 43.86[16.89%] | 4.82 |
|  | **100** |  |  | 46.12[9.00%] | 5.22 |
|  | **120** |  |  | 54.20[10.00%] | 6.16 |
| **4 to 6**  **[17.34-23.81]** | **10** | 6.15 [10.63 %] | 0.18 | 4.46 [13.57%] | 0.42 |
|  | **20** | 13.43 [8.8%] | 0.94 | 8.75 [12.41%] | 1.02 |
|  | **30** | 20.42[14.67%] | 0.64 | 13.08[8.75%] | 1.17 |
|  | **40** | 24.27[11.93%] | 0.91 | 17.39[13.98%] | 1.58 |
|  | **50** | 35.85[12.11%] | 0.81 | 23.55[10.94%] | 2.40 |
|  | **60** | 37.14[10.57%] | 0.78 | 27.15[9.92%] | 3.07 |
|  | **70** | 47.37[12.60%] | 0.98 | 29,72[10.83%] | 3.15 |
|  | **80** | 55.94[11.11%] | 1.13 | 32.86[14.18%] | 3.37 |
|  | **90** |  |  | 40.27[15.35%] | 3.50 |
|  | **100** |  |  | 44.98[9.91%] | 4.67 |
|  | **120** |  |  | 46.54[22.15%] | 6.70 |
| **7 to 9**  **[26.54-34.45]** | **10** | 7.35[9.96%] | 0.98 | 4.74[14.46%] | 0.32 |
|  | **20** | 14.63[11.56%] | 1.11 | 8.72[11.49%] | 1.51 |
|  | **30** | 19.83[6.56%] | 1.14 | 14.29[11.03%] | 2.00 |
|  | **40** | 26.46[14.98%] | 1.19 | 18.19[11.24%] | 2.45 |
|  | **50** | 26.85[10.49%] | 1.83 | 28.96 [10.55] | 2.70 |
|  | **60** | 40.07[9.52%] | 1.66 | 29.53[14.02%] | 4.46 |
|  | **70** | 43.53[12.92%] | 1.98 | 34.52[15.72%] | 4.59 |
|  | **80** | 53.75[13.29%] | 2.51 | 37.40[9.99%] | 4.61 |
|  | **90** |  |  | 43.12[11.41%] | 5.72 |
|  | **100** |  |  | 45.56[13.04%] | 5.63 |
|  | **120** |  |  | 48.53[10.05%] | 10.5 |

Table S3. Simulated mean maximum concentration (C_max_), steady-state plasma concentration range, and their corresponding coefficient of variation (CV%) in the pediatric population

| **Age (years) [weight] (kg)** | **Dose**  **(mg kg^-1^ day^-1^)** | **BID** | | **TID** | |
| --- | --- | --- | --- | --- | --- |
|  |  | **Cmax**  **(µg mL^-1^)** | **Range**  **[min and max]**  **(µg mL^-1^)** | **Cmax**  **(µg mL^-1^)** | **Range [min and max]**  **(µg mL^-1^)** |
| **0.5 to 3 [8.29-14.95]** | **10** | 6.78 [13.24 %] | 4.68-7.58 | 4.59 [11.62%] | 3.72-5.57 |
|  | **20** | 13.71 [9.67%] | 10.75-15.02 | 8.32 [14.38%] | 7.04-11.88 |
|  | **40** | 26.11 [11.27%] | 19.03-31.56 | 18.58 [9.32%] | 15.58-20.52 |
|  | **60** | 38.81 [32.03%] | 37.02-45.31 | 27.27[13.14%] | 22.48-30.05 |
|  | **80** | 48.93[6.92%] | 42.38-53.46 | 39.5[13.75%] | 25.72-45.30 |
|  | **90** |  |  | 43.86[16.89%] | 26.69-50.42 |
|  | **100** |  |  | 46.12[9.00%] | 44.46-54.15 |
|  | **120** |  |  | 54.20[10.00%] | 41.18-60.48 |
| **4 to 6 [17.34-23.81]** | **10** | 6.15 [10.63 %] | 4.93-6.88 | 4.46 [13.57%] | 3.41-5.65 |
|  | **20** | 13.43 [8.8%] | 10.5-15.56 | 8.75 [12.41%] | 6.82-10.7 |
|  | **40** | 24.27 [11.93%] | 18.34-27.36 | 17.39[13.98%] | 12.95-21.19 |
|  | **60** | 37.14 [10.57%] | 25.56-39.51 | 27.15[9.92%] | 21.51-30.06 |
|  | **80** | 55.94 [11.11%] | 39.37-56.82 | 32.86[14.18%] | 20.93-37.34 |
|  | **90** |  |  | 40.27[15.35%] | 28.08-49.49 |
|  | **100** |  |  | 44.98[9.91%] | 34.79-49.15 |
|  | **120** |  |  | 46.54[22.15%] | 33.27-69.29 |
| **7 to 9**  **[26.54-34.45]** | **10** | 7.35[9.96%] | 6.02-8.51 | 4.74[14.46%] | 4.50-5.68 |
|  | **20** | 14.63[11.56%] | 14.15-16.45 | 8.72[11.49%] | 6.59-10.55 |
|  | **40** | 26.46[14.98%] | 18.33-30.52 | 18.19[11.24%] | 14.64-21.36 |
|  | **60** | 40.07[9.52%] | 33.14-47.63 | 29.53[14.02%] | 18.21-33.72 |
|  | **80** | 53.75[13.29%] | 35.48-61.83 | 37.40[9.99%] | 28.52-42.19 |
|  | **90** |  |  | 43.12[11.41%] | 34.16-49.32 |
|  | **100** |  |  | 45.56[13.04%] | 32.35-52.23 |
|  | **120** |  |  | 48.53[10.05%] | 39.2-68.37 |
| **10 to 12**  **[38.9-47.68]** | **10** | 7.35[9.96%] | 6.02-8.51 | 5.42[14.74%] | 5.05-6.19 |
|  | **20** | 14.63[11.71%] | 11.56-16.45 | 10.56[9.64%] | 8.89-12.28 |
|  | **40** | 29.37[10.04%] | 22.40-33.36 | 16.42[13.89%] | 11.84-19.6 |
|  | **60** | 44.09[12.08%] | 33.52-53.78 | 35.52[12.5%] | 30.38-36.46 |
|  | **80** | 58.05[7.97%] | 50.39-67.97 | 42.15[13.09%] | 30.03-44.75 |
|  | **90** |  |  | 49.03[11.41%] | 36.55-55.71 |
|  | **100** |  |  | 54.41[16.92%] | 35.92-67.54 |
|  | **120** |  |  | 64.88[9.35%] | 46.56-68.32 |

BID: twice a day administration; TID: three times a day administration.

**
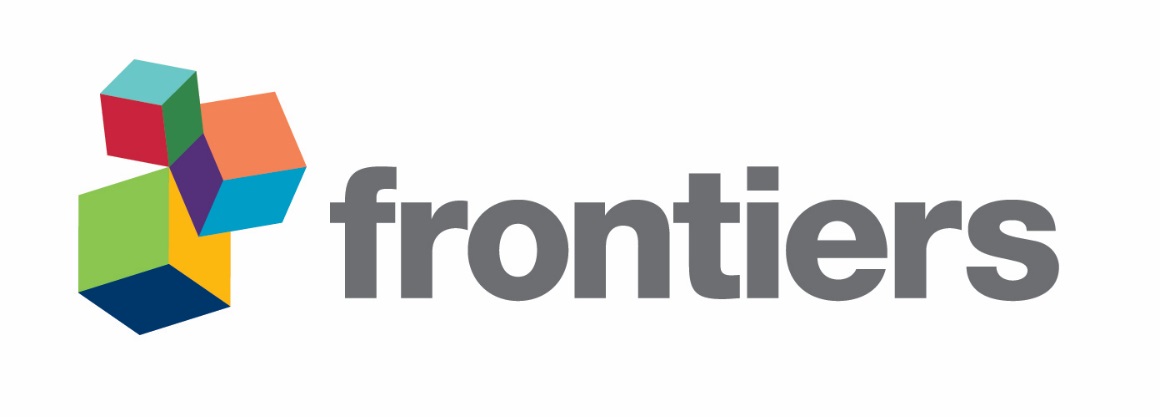
**
